# Identification of the xyloglucan endotransglycosylase/hydrolase genes and the role of *PagXTH12* in drought resistance in poplar

**DOI:** 10.48130/forres-0024-0036

**Published:** 2024-12-31

**Authors:** Wenya Yuan, Fengge Yao, Yijing Liu, Hongci Xiao, Siheng Sun, Cheng Jiang, Yi An, Ningning Chen, Lichao Huang, Mengzhu Lu, Jin Zhang

**Affiliations:** State Key Laboratory of Subtropical Silviculture, College of Forestry and Biotechnology, Zhejiang A&F University, Hangzhou 311300, Zhejiang, China

**Keywords:** *Populus*, Xyloglucan endotransglycosylase/hydrolase, Paralogous pair, Drought stress

## Abstract

The xyloglucan endotransglycosylase/hydrolase (XTH) gene family plays a crucial role in plant cell wall remodeling, facilitating growth and structural changes. However, the divergence of paralogous genes among different species of *Populus* remains inadequately understood. This study investigates the phylogenetic relationships and expression characteristics of *XTH* genes in two *Populus* species: *Populus trichocarpa* and *Populus alba × P. glandulosa* '84K'. Forty-one *XTHs* were identified in *P. trichocarpa* and 38 and 33 members in the subgenome A and G of '84K' poplar, respectively. Gene expression analysis demonstrated differences among paralogous genes within the same subgenome and between orthologous genes across species, likely influenced by variations in promoter regions. Notably, *XTH12* showed a specific response to drought stress among various abiotic stresses. In a population of 549 *Populus* individuals, functional SNPs in *XTH12*'s coding region did not affect its conserved ExDxE catalytic site, highlighting its irreplaceable function. Furthermore, validation through qRT-PCR and *ProPagXTH12::GUS* activity, alongside *PagXTH12*-overexpression poplar lines, substantiated the role of PagXTH12 in modulating the balance between plant biomass and drought resistance. Overall, this research provides valuable insights into the biological functions of XTHs in plant environmental adaptability and offers strategies for targeted regulation of tree growth and stress resistance.

## Introduction

Cell walls, the robust matrices that define plant cellular morphology and provide mechanical support, are primarily composed of cellulose, lignin, hemicellulose, pectin, and various minor constituents. Of these components, xyloglucan is a pivotal hemicellulose component found predominantly in the primary cell walls of dicotyledons^[1]^. Beyond providing shape and structural integrity, cell walls play essential roles in growth and developmental processes^[2]^. The dynamic regulation of cell wall composition and architecture involves a multitude of molecules and biological processes. Among these, cell wall-loosening proteins are particularly important for enhancing the plasticity and extensibility of the cell wall. Xyloglucan endotransglucosylase/hydrolase (XTH) enzymes are key players in this process, facilitating either the hydrolysis or transfer of xyloglucan molecules, as well as the cleavage or rearrangement of the xyloglucan backbone^[3]^. These actions significantly contribute to the elongation of plant cell walls.

XTH belongs to the glycoside hydrolase family 16 (GH16) and exhibit dual functionalities as xyloglucan endoglycosidases (XEH) and xyloglucan hydrolases (XET)^[4]^. The glutamic acid residue located in the active site of xyloglucan internal glycosyltransferase/hydrolase not only catalyzes the hydrolysis of xylan chains, but also facilitates the transfer and rejoining of these chains^[5]^. The amino acid sequence of XTH contains a conserved catalytic motif, DEIDFEFLG, which has been identified as the active site for both hydrolases and glycosyltransferases^[6]^. Moreover, this sequence includes a signal peptide that aids in the secretion of the enzyme into the apoplast^[7]^. Based on phylogenetic relationships, XTH genes are classified into three groups (I, II, and III), with group III further subdivided into subgroups IIIA, IIIB, and an early diverging group^[8]^.

Recent advancements in molecular biology and bioinformatics have facilitated the identification and functional characterization of the *XTH* gene family across various plant species. To date, 33, 29, and 61 *XTH* genes have been identified in *Arabidopsis thaliana*^[7]^, *Oryza sativa*^[9]^, and *Glycine max*^[10]^, respectively. These *XTH* genes are implicated in crucial processes related to plant growth and development. For instance, in *Arabidopsis*, overexpression of *AtXTH22* has been shown to enhance cell wall thickness and promote primary root growth^[11]^. In maize, *ZmXTH1* is involved in modulating cell wall composition and structure^[12]^. Moreover, in persimmons, overexpression of *DkXTH1* increases tolerance to abiotic stress and delays fruit softening^[13]^. The study of genomics will contribute to further research on tree growth and development, thereby assisting in forest tree breeding^[14]^.

Poplar (*Populus* L.), are recognized for their rapid growth, carbon sequestration potential, and environmental remediation capabilities, rendering them ecologically and economically valuable^[15]^. The relatively small genome of poplar and the availability of established transgenic systems makes it an exemplary model for genetic, taxonomic, and evolutionary studies^[16]^.

In this study, members of the *XTH* family within the genome of *Populus trichocarpa* and a hybrid poplar (*Populus alba* × *P. Glandulosa*, '84K') were identified and classified. A comprehensive analysis of their evolutionary relationships, gene duplication events, gene structures, *cis*-acting elements, and expression patterns across different tissues and under various stress conditions were investigated. The systematic investigation revealed characteristics related to the structure, function, and evolution of the *XTH* gene family in poplar, laying a theoretical foundation for further exploration of the potential functions of *XTH* genes in poplar. Additionally, the protein structure and enzymatic activity of PagXTH12 were analyzed, and the overexpression of *PagXTH12* in poplar confirmed its role in regulating the balance between plant biomass and drought resistance. The present results provide new insights into the potential roles of *PagXTH12* genes in drought stress responses.

## Materials and methods

### Identification of *PagXTHs*

The nucleotide and protein sequences of the XTH family in Arabidopsis were downloaded from the TAIR website (www.arabidopsis.org/index.jsp). The nucleotide and protein sequences of the XTH family in *P. trichocarpa* version 4.1 were downloaded from the Phytozome website (https://phytozome-next.jgi.doe.gov/). The XTH family members in *Populus alba* × *P. glandulosa* '84K' poplar were obtained by BLAST with an *E*-value of 1e-10 with the protein sequences of *P. trichocarpa* and Arabidopsis*.*

### Phylogenetic analyses

Interspecific phylogenetic tree (*Populus trichocarpa*, *Populus alba* × *P. glandulosa* '84K', and Arabidopsis) and intraspecific phylogenetic tree of '84K' poplar were subsequently constructed by the maximum likelihood (ML) method with 100 bootstrap replications^[17]^. The phylogenetic trees were then visualized using the online tool Evolview v3 (www.evolgenius.info/evolview)^[18]^.

### Gene structure and motif analyses

The exon-intron structural diversity was analyzed by the online Gene Structure Display Server (GSDS) (http://gsds.cbi.pku.edu.cn/; v2.0)^[19]^. The structural maps of the *PagXTHs* were ultimately mapped to the intraspecific phylogenetic tree. To identify additional conserved motifs outside the XTH domain, the protein sequences were analyzed by the online MEME server (http://meme-suite.org/; v5.1.1), and the number of motifs was set to 20 (*E*-value < 0.0001)^[20]^. The gene structure, motifs, and conserved domain were visualized by TBtools^[21]^.

### Analysis of promoter *cis-*acting elements and promoter sequence differences

The promoter sequences of *XTHs* (2 kb upstream of translation initiation site) were analyzed for *cis*-acting elements analysis, using the PlantCARE database (http://bioinformatics.psb.ugent.be/webtools/plantcare/html/)^[22]^. The computationally predicted *cis*-acting elements were then divided into three categories (stress responses, hormone responses, and development) according to their biological functions. The similarity of the promoters between the *XTH* paralogous pair was analyzed using the Cross Species function of PlantPAN 3.0 (http://plantpan.itps.ncku.edu.tw/plantpan3/cross_species.php)^[23]^.

### Genome-wide duplication of *XTHs* in *Populus*

The internal and intergenic gene repeat patterns and collinearity relationships of *PtrXTHs* and *PagXTHs* in the genome were identified and analyzed using the MCScanX software^[24,25]^. Subsequently, visualization analysis was conducted using the Circos software^[26]^.

### Gene expression and co-expression network of *PtrXTHs*

The expression data of *XTH* genes in various tissues and under drought treatment were obtained from the *Populus* Gene Atlas Study (https://phytozome-next.jgi.doe.gov/), the NCBI Bioprojects (PRJNA526157 and PRJNA736374), and the EBI database (accession number: PRJEB19784). Co-expression relationships of *PtrXTHs* were downloaded from Phytozome (https://phytozome.jgi.doe.gov/pz/portal.html#)^[27]^. Genes with a Pearson Correlation Coefficient (PCC) ≥ 0.85 and *p* < 0.05 were selected for co-expression network construction, using a significance threshold. The co-expression network was visualized using Cytoscape software^[28,29]^.

### Gene and promoter cloning and construction of vector

RNA was extracted from '84K' poplar tissue culture seedlings, followed by cDNA synthesis through reverse transcription. The full-length coding sequence of *PagXTH12(A)* measuring 876 bp was cloned and inserted into the pMDC32 and pMDC43 vectors using the Gateway method for overexpression and subcellular localization, respectively. Both vectors use the 35S promoter to drive high-level expression of *PagXTH12* in plant tissues. Additionally, a 2,200 bp upstream promoter region of the *PagXTH12(A)* start codon was cloned and inserted into the pMDC164 vector for Pro*PagXTH12::GUS* construct using the Gateway method.

### Agrobacterium mediated genetic transformation

The genetic transformation in this experiment was conducted using Agrobacterium-mediated callus transformation, encompassing the following steps. The leaves from the 3^rd^ to 5^th^ leaves of the 3-week-old tissue-cultured poplars were placed on callus-inducing medium for dark induction of callus. The induced '84K' poplar callus tissues were then immersed in Agrobacterium liquid (OD = 0.6) for 15 min. Subsequently, they were placed on shoot-inducing medium to induce differentiation into adventitious shoots. After screening on a medium containing antibiotics, adventitious shoots were induced to root. Finally, transgenic plants were identified through PCR using DNA extracted from leaves as a template^[30]^.

### Analysis of PagXTH12 protein structure and activity

Protein structure prediction was conducted using the AlphaFold3 webserver (https://alphafoldserver.com). The single nucleotide polymorphisms (SNPs) in the *XTH* genes were obtained from Phytozome, which was based on the whole genome re-sequencing data of 549 *P. trichocarpa* natural individuals in North America^[31]^. Enzyme activity within the plant was measured using enzyme-linked immunosorbent assay (ELISA), specifically with the Plant Xyloglucan Endotransglucosylase/Hydrolase (XTH) ELISA Kit (MM-1720O2).

### Drought treatment

3-week-old Pro*PagXTH12::GUS* seedlings were sequentially exposed to a 20% PEG6000 solution for 0, 3, and 6 h to evaluate the osmotic stress response. To further investigate the drought stress response of the *PagXTH12* gene, 3-month-old wild-type (WT) and transgenic plants overexpressing *PagXTH12* grown in a greenhouse were subjected to natural drought treatment (withholding irrigation), with six biological replicates per treatment group. Observations were conducted on the 10^th^ and 15^th^ days following the initiation of drought treatment.

### GUS staining

Seedlings were fixed with 90% acetone and placed at 4 °C or on ice for 2 h. After rinsing the treated plants with GUS (β-glucuronidase) staining buffer at least three times, they were immersed in GUS staining solution. Following this, the plants underwent vacuum infiltration for 30 min and were then placed in an incubator at 37 °C for 10 h for staining. Finally, decolorization was performed using 75% ethanol. The whole seedlings' staining results were observed using a stereomicroscope (Stereo D13covery V12). Three biological replicates were used for different treatment stages.

### Subcellular localization

The subcellular localization of the PagXTHs were predicted by the online tool CELLO (http://cello.life.nctu.edu.tw/; v2.5)^[32]^. The full-length coding sequence of PagXTH12(A) was cloned into the pMDC43 vector *via* Gateway recombination technology. Agrobacterium strain GV3101 harboring this recombination vector was transfected into 3-week-old *Nicotiana benthamiana*. Confocal microscopy was performed using a Zeiss LSM 880 laser scanning microscope.

### Quantitative RT-PCR

The total RNA from the samples was extracted using the RNA-prep Pure Plant Plus Kit (Tiangen, China). Subsequently, the total RNA underwent reverse transcription using the Evo M-MLV Reagent Kit and gDNA Eraser (AGbio, China). RT-qPCR was conducted using the SYBR Green Pro Taq HS Reagent Kit (AGbio, China). *ACTIN* (Potri.001G309500) served as the internal control gene. The RNA samples used were three biological replicates. The RT-PCR experiments were conducted with three technical replicates. All the primers used in this study are listed in Supplementary Table S1.

### Statistical analysis

Statistical analysis to determine statistical significance was performed by Student's *t*-tests for paired samples or one-way ANOVA followed by Tukey's post hoc test for multiple pairwise comparisons.

## Results

### Identification and phylogenetic analyses of *XTH* genes in two poplar species

Previous studies reported the presence of 43 *XTH* genes in the poplar genome^[33]^. However, with the continuous improvement of woody plant genomes and the assembly of more gap-free genomes, the identification of functional genes has become more accurate. In this study, the latest genomic versions of two poplar species were utilized, *Populus trichocarpa* and *Populus alba* × *P. glandulosa* '84K', to compare the members of the *XTH* gene family within poplars. According to the most recent version of the *P. trichocarpa* genome (V4.1), it was found that *PtrXTH11*, which was originally annotated on chr4 in an earlier genome version (V3.0), as well as *PtrXTH41* and *PtrXTH42* on scaffold_174, does not exist. Moreover, *PtrXTH43*, previously located on scaffold_2348, should be situated on chr5 (Potri.005G201250). Additionally, a new, unreported *XTH* gene on chr9 was discovered, which was named *PtrXTH44* (Potri.009G163850). Therefore, based on the latest genomic version, *P. trichocarpa* contains a total of 41 *XTH* genes (Supplementary Table S2).

To further analyze the evolutionary relationships of *XTH* genes among different species of poplar, the evolutionary conservation of the *XTH* family in the hybrid poplar 84K was investigated. This analysis is facilitated by the availability of complete genomic information from both parents (subgenome A from *P. alba* and subgenome G from *P. tremula* var. *glandulosa*), enhancing our understanding of the evolutionary dynamics of *XTH* genes within the poplar lineage. Interestingly, through a comprehensive genome search, varying degrees of *XTH* gene loss in both subgenomes of '84K' poplar were identified. Specifically, three *XTH* genes (*XTH19/27/33*) were absent from both subgenomes, whereas five genes (*XTH12/25/26/39/43*) were present exclusively in subgenome A and missing in subgenome G (Supplementary Table S2). These findings suggest that the lost genes may exhibit functional redundancy or possess unique biological functions specific to different poplar species.

A phylogenetic tree was susequently constructed using the identified 41 *PtrXTH* genes, 38 *PagXTH(A),* and 33 *PagXTH(G)* genes from the two subgenomes of '84K' poplar, and 33 *AtXTH* members from Arabidopsis. Consistent with previous reports on the grouping of XTH family members, the XTH family members from these two poplar species can also be categorized into four distinct groups: Group I/II, Group IIIA, Group IIIB, and Early Diverging Group (Supplementary Fig. S1). Among the four groups, Group I/II contains the greatest number of members and exhibits the largest variation in sequence length. The amino acid lengths of the protein sequences in this group range from 208 to 518, with molecular weights between 2.40 and 5.96 kDa. The early diverging group comprises only one member from poplar (XTH7), which shows a high conservation in both the genomic sequences of *P. trichocarpa* and two subgenomes of '84K' poplar, consisting of 289 amino acids. Groups IIIA and IIIB contain three and six poplar XTH members, with amino acid lengths ranging from 236 to 312 and 170 to 370, respectively (Supplementary Table S3).

The subcellular localization of proteins is crucial to their functional roles. Through the prediction of subcellular localization for XTH members from two poplar species, it was found that nearly all XTH members exhibit potential for localization in the cell wall, while some may also localize in the cytoplasm (Supplementary Table S3). This suggests that the localization of XTH members within plant cells may be dynamic. Furthermore, specific XTH members demonstrate localization differences between the two subgenomes of '84K' poplar (PagXTH16/18/20/29/32/35/38), which may result from sequence variations in the alleles.

### Gene structure and conserved motif analyses

**Figure 1:**
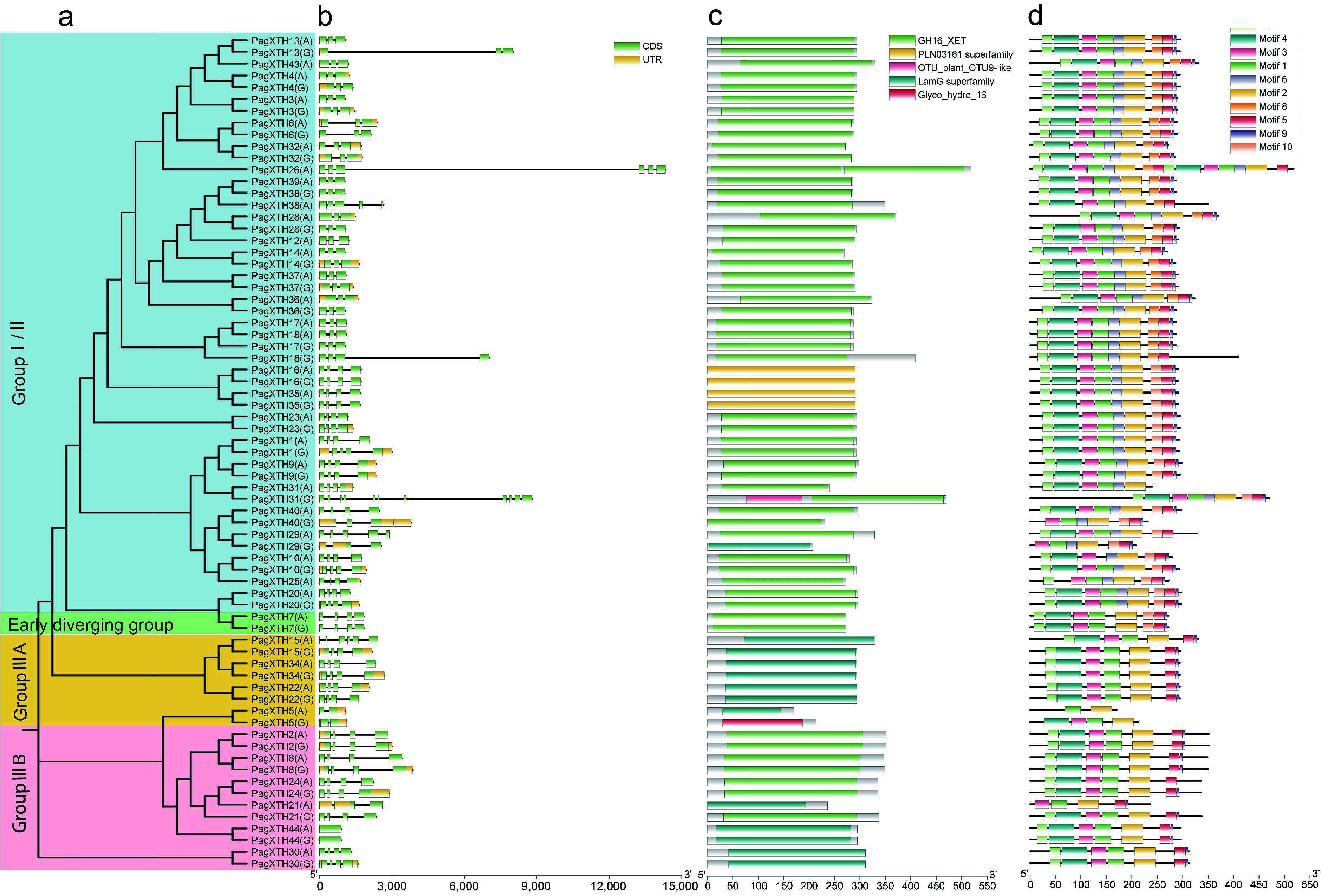
Analysis of gene structure, protein domains, and conserved motifs in the *XTH* gene family in poplar. (a) Phylogenetic tree of PagXTHs the maximum likelihood (ML) method with 100 ultrafast bootstrap replications. (b) Gene structures of *PagXTHs*. Exons, introns, and UTR regions are represented by green rectangles, gray lines, and yellow rectangles, respectively. (c) Colored rectangular blocks illustrate the conserved domains within the XTH proteins. (d) Conserved motifs identified by MEME, different colored blocks represent various motifs.

To further elucidate the evolutionary relationships among the XTH family members in poplar, an analysis of the gene structures and conserved domains of XTH members within the two subgenomes of the '84K' poplar was conducted (Fig. 1a). The findings indicate that although the majority of allelic structures across both subgenomes are similar, certain alleles exhibit discrepancies in gene structure and conserved regions. For instance, three genes (XTH13/18/31) in subgenome G possess longer introns compared to their alleles in subgenome A, which exhibit shorter intron lengths (Fig. 1b). Notably, the conserved domain of PagXTH31(G) contains an additional OTU9-like domain compared to its allele PagXTH31(A) (Fig. 1c). In contrast, *XTH26* is exclusively found in subgenome A and also features long introns. Domain analysis reveals that PagXTH26(A) includes two GH16_XET domains, implying a structural configuration resembling the fusion of two distinct XTH gene structures. However, it remains indeterminate whether this duplicated sequence segment originated from subgenome G. Furthermore, the newly identified *XTH44* gene lacks introns altogether. Among the identified conserved motifs (Fig. 1b, c), motifs 1, 3, 4, 6, and 7 correspond to the GH16 domain. Notably, motif 3 was detected in all XTH family members and encompasses the conserved sequence of the active site characteristic of XTH family proteins: DEIDFEFLG (Fig. 1d). Among these sequences, the ExDxE motif has been demonstrated to serve as an active catalytic site.

### Genome-wide duplication of *XTH*s in *Populus*

**Figure 2:**
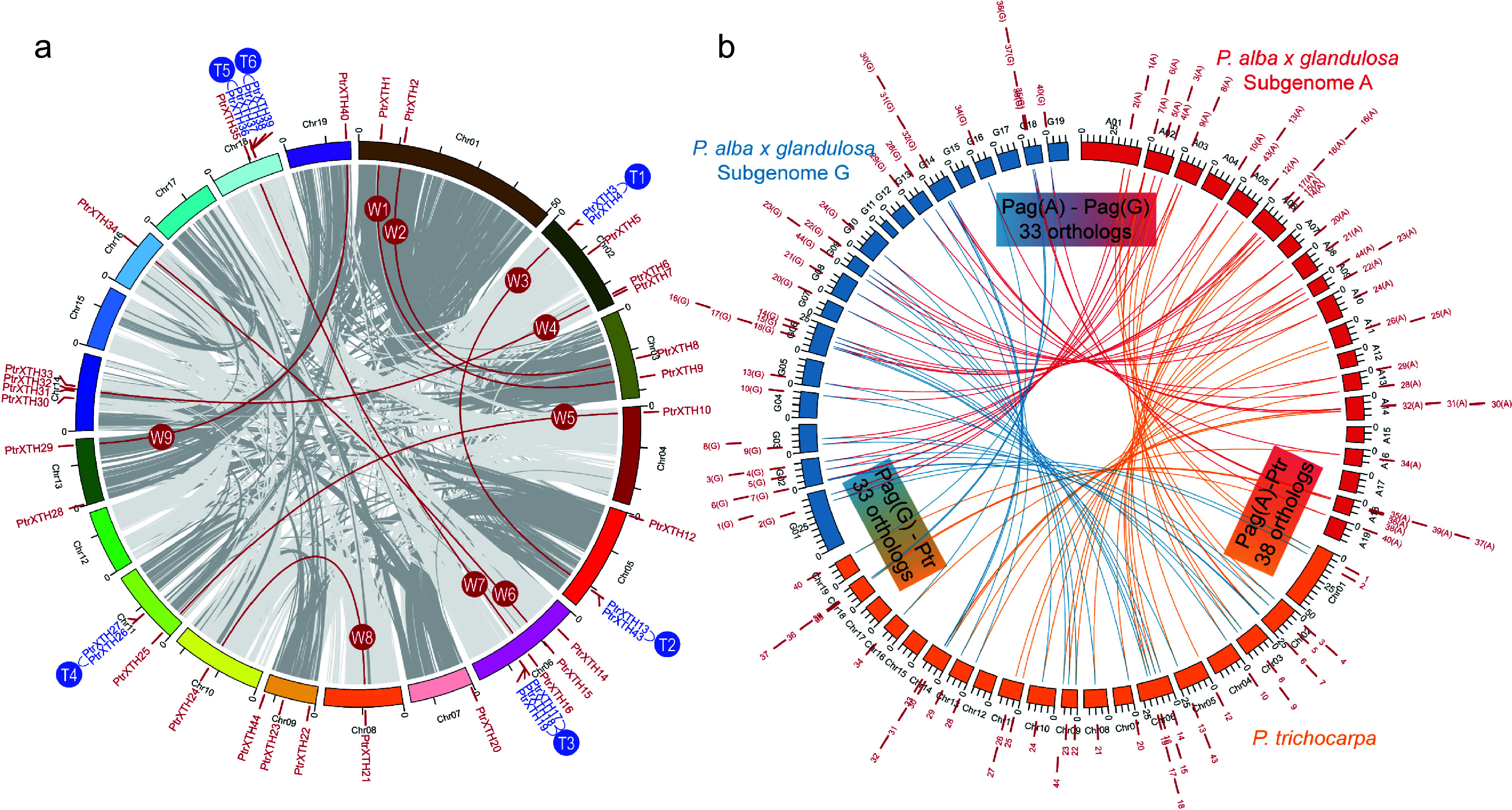
Genomic location and gene duplication of the *XTH* genes in the poplar genome. (a) Genomic location and gene duplication of the *XTH* genes in the *P. trichocarpa* genome. The XTH paralogous pairs were generated by the whole-genome duplication events (W1−W9) or tandem duplication events (T1−T6). (b) Collinearity analysis reveals the orthologous relationship of *XTH* genes between the genome of *P. trichocarpa* and two subgenomes (A and G) of *Populus alba* × *glandulosa* '84K'.

To further investigate the inter- and intra-specific evolutionary relationship between *PtrXTHs* and *PagXTHs*, a collinearity analysis of the *XTH* families across *P. trichocarpa* and two subgenomes of '84K' poplar were conducted (Fig. 2). In the *P. trichocarpa* genome, a total 41 *PtrXTH* genes were distributed across 15 of the 19 chromosomes. Among these, nine pairs of paralogous genes (W1−W9) were generated through whole-genome duplication events, while six pairs (T1−T6) arose from tandem duplication events (Fig. 2a & Supplementary Table S4). In the case of the two subgenomes of '84K' poplar, the gene count has decreased due to the absence of certain *XTH* members. Subgenome A contains 38 orthologous genes corresponding to *P. trichocarpa* XTHs, whereas subgenome G harbors 33 such orthologs (Fig. 2b), indicating that subgenome A of '84K' poplar is evolutionarily closer to *P. trichocarpa*. Overall, there are 33 alleles of *XTH* in the two subgenomes of '84K' poplar, with five *XTH* members unique to subgenome A potentially exerting gene dosage effects on the hybrid poplar.

### Analysis of conserved sequences and *cis*-acting elements in the promoter of the *XTH* gene in poplar

**Figure 3:**
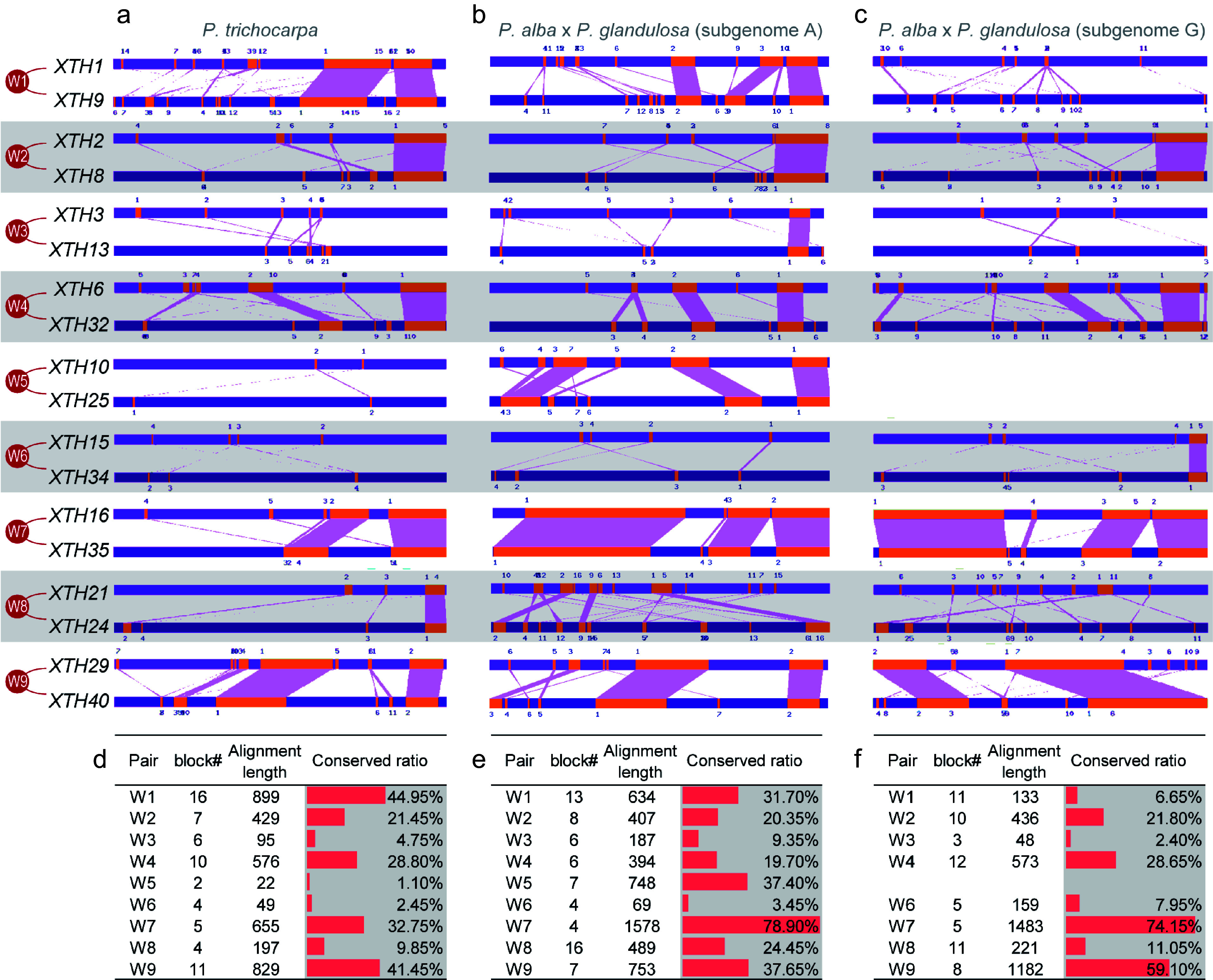
The promoter similarity between paralogous pairs of *PtrXTHs* and *PagXTHs*. (a)−(c) Conserved blocks located in the promoter region of the *XTH* paralogous pairs in *P. trichocarpa* genome, *Populus alba* × *glandulosa* subgenome A, and subgenome G, respectively. (d)−(f) Conserved block number, alignment length and repetition rate between the promoter of *XTH* paralogous pairs in *P. trichocarpa* genome, *Populus alba* × *glandulosa* subgenome A, and subgenome G, respectively.

In alleles, the loss or structural variation of genes not only affects gene function but differences in expression between alleles and paralogous homologs can also significantly influence their functional roles in specific tissues or under certain environmental conditions. Gene expression is directly regulated by promoter sequences and their cis-acting elements. Based on the collinearity analysis of the *XTH* family, the promoter similarity of *PtrXTH* and *PagXTH* paralogous gene pairs (W1−W9) were compared (Fig. 3a−c). The results indicate significant differences in the promoter similarity of paralogous pairs between *P. trichocarpa* and the two subgenomes of '84K' poplar. For example, the promoters of gene pair W5 exhibited the lowest similarity in *P. trichocarpa* (1.10%), while the similarity for this gene pair rose to 37.40% in subgenome A of the '84K' poplar. In contrast, gene pair W7 demonstrated the highest promoter similarity in the '84K' poplar (78.90% in subgenome A and 74.15% in subgenome G), but it only reached 32.75% similarity in *P. trichocarpa*. Additionally, there is substantial variation in promoter similarity among different paralogous gene pairs within the various subgenomes of the '84K' poplar. For instance, gene pair W1 showed significantly higher similarity in subgenome A (31.70%) compared to subgenome G (6.65%) (Fig. 3d−f).

The regulatory *cis*-acting elements and transcription factor binding sites within the promoter regions play a pivotal role in modulating gene expression. Through statistical analysis, the *cis*-acting elements of the *XTH* gene family were categorized into three functional groups: stress-responsive, hormone-responsive, and growth and development-responsive elements (Supplementary Fig. S2a). The expression of the *XTH* gene family in '84K' poplar is likely influenced by various external stresses and internal hormone levels, while also participating in plant growth and developmental processes. Among these elements, the stress-responsive elements, such as STRE, ABA-responsive element (ABRE), as-1, estrogen response element (ERE), ANAEROBIC RESPONSE ELEMENT (ARE), were found in relatively high abundance. Notably, *PagXTH12(A)*, *PagXTH13(A)*, and *PagXTH17(A)* exhibited the highest occurrence of STRE elements, each containing 11 copies, suggesting their significant potential in stress response. Specifically, *PagXTH12(A)* includes one ABRE, one MYB binding site (MBS), and two dehydration-responsive element (DRE) elements, all of which are associated with drought stress (Supplementary Fig. S2b).

### Tissue-specific expression patterns of *PtrXTHs* and *PagXTHs*

**Figure 4:**
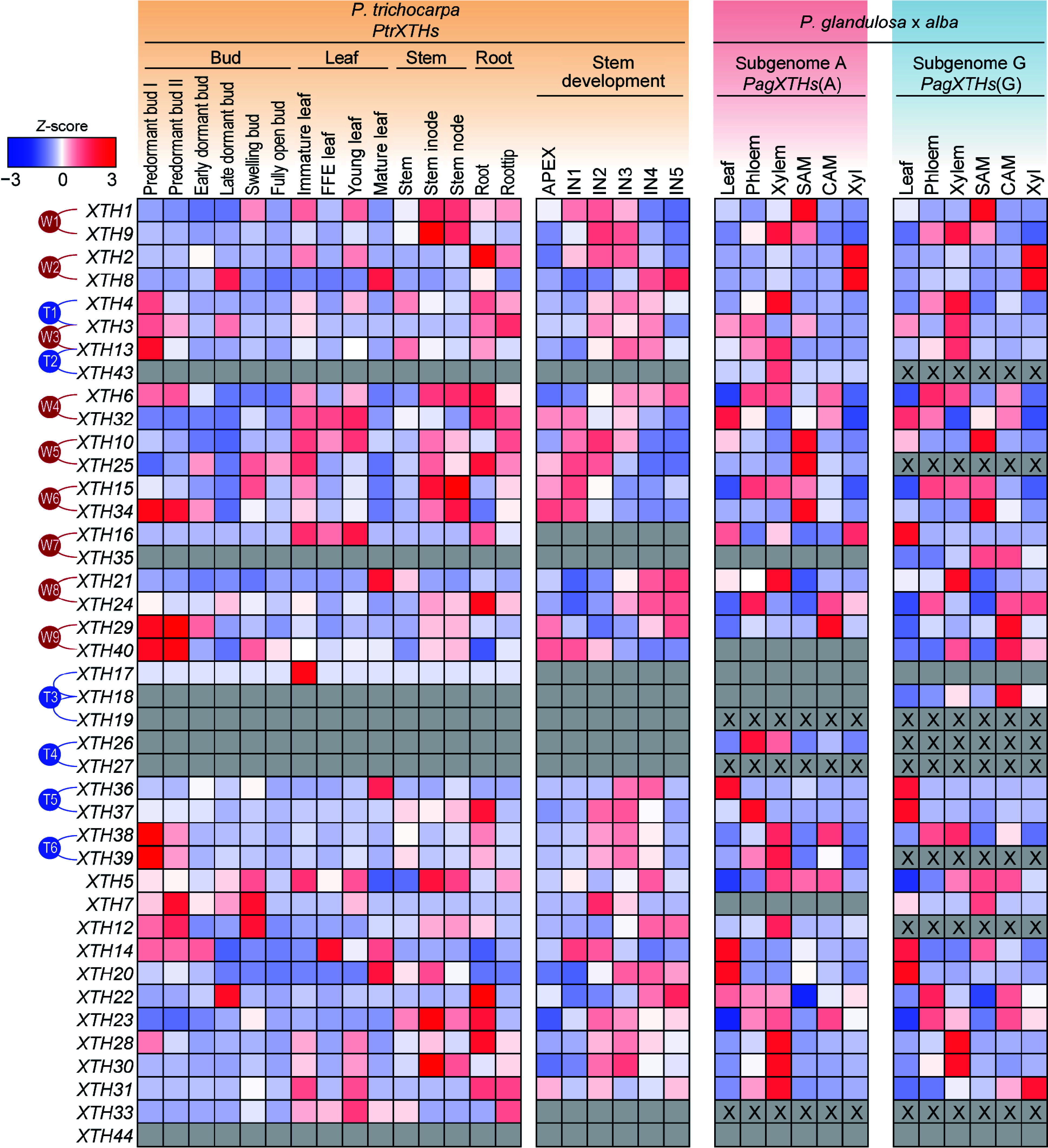
The expression patterns of *PtrXTHs* and *PagXTHs* across various tissues. The expression data including bud, leaf, and stem were obtained from *Populus* Gene Expression Atlas and NCBI Bioprojects (PRJNA526157 and PRJNA736374). Gene expression was normalized by *Z*-score. Blue and red represent low and high expression, respectively. The gray boxes indicate that gene expression was not detected, and 'X' within the box represent the absence of a corresponding *XTH* gene in this subgenome. The paralogous genes generated by the whole-genome duplication events (W1−W9) or the tandem duplication events (T1−T6) are marked with red or blue lines on the left.

Furthermore, the expression patterns of the *XTH* gene family in different tissues of *P. trichocarpa* and '84K' poplar were analyzed. Generally, paralogous gene pairs that have diverged more recently exhibit similar expression patterns. In these two poplar species, there are some differences among the paralogous gene pairs. For instance, the expression patterns of the two genes, *XTH1* and *XTH9*, in the paralogous pair W1 show a consistent trend across various tissues in *P. trichocarpa*; however, this trend is inconsistent in '84K' poplar, where the two alleles of *XTH1* are expressed at higher levels in the shoot apical meristem, while the two alleles of *XTH9* show higher expression in the xylem. In contrast, the two genes, *XTH2* and *XTH8*, in the paralogous pair W2 exhibit similar expression trends in '84K' poplar, but in *P. trichocarpa*, *XTH2* is predominantly expressed in the roots and primary stems, whereas *XTH8* shows higher expression in the leaves and secondary stems. Additionally, some gene pairs have members where only one allele is expressed in hybrid poplar; for example, in the paralogous pair W9, *XTH40* was only detected in subgenome G (Fig. 4).

### Expression patterns of *XTH* genes under abiotic in poplar

**Figure 5:**
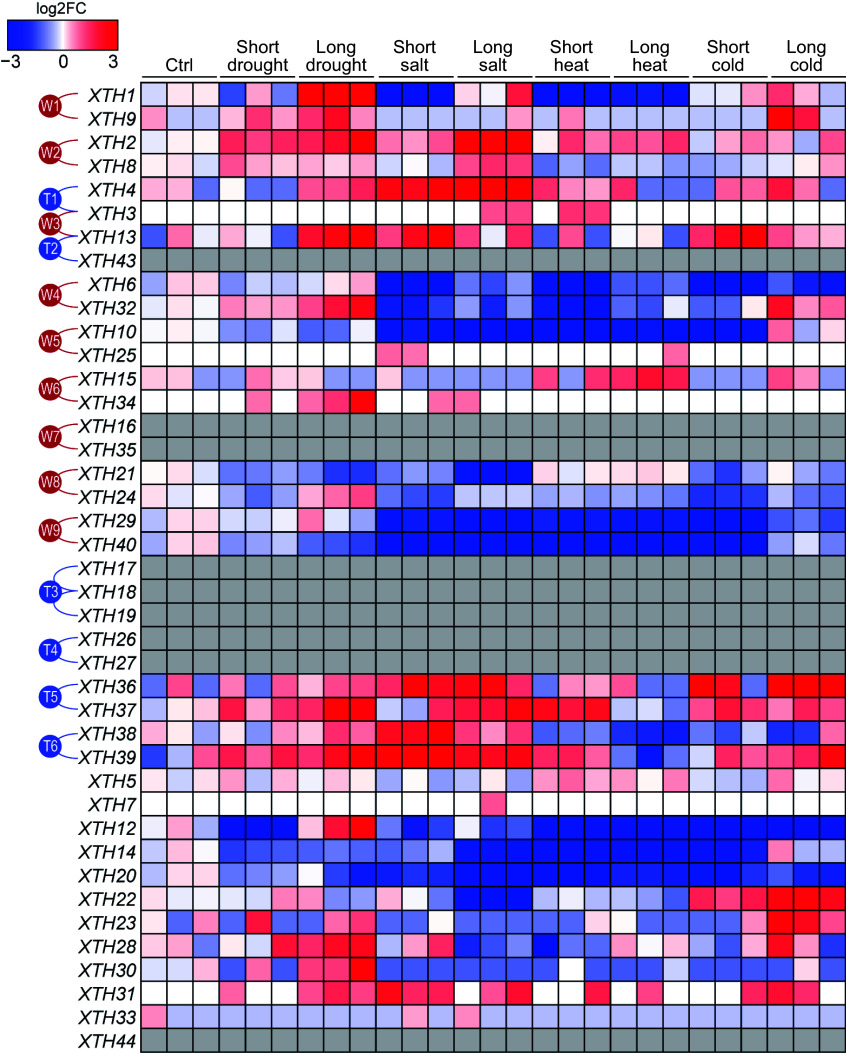
The expression patterns of *PtrXTHs* under various abiotic stress conditions. The gene expression data, including control conditions (Ctrl) and short-term and long-term treatments under drought, salt stress, high temperature, and low temperature, were obtained from the EBI database (accession number: PRJEB19784). Gene expression was normalized by log2 fold change compared to the control. Blue and red represent low and high expression, respectively. The gray boxes indicate that gene expression was not detected. The paralogous genes generated by the whole-genome duplication events (W1−W9) or the tandem duplication events (T1−T6) are marked with red or blue lines on the left.

When comparing the expression profiles of *XTHs* under various abiotic stress conditions, such as drought, salinity, high temperature, and low temperature, it was observed that different *XTHs* exhibit diverse expression patterns. This suggests that they may play significant roles in the plant's adaptation to specific environmental conditions. As illustrated in Fig. 5, certain paralogous gene pairs within the *XTH* family respond to multiple stresses; for instance, members of paralogous pairs W1, W2, and W3 respond to both drought and salt stress, while members of T5 and T6 respond to drought, salt, and temperature stress. However, the degree of response among members within the same paralogous pairs varies. For example, *XTH2* from the paralogous pair W2 shows a significantly stronger response to stress compared to *XTH8*, and *XTH13* from pair W3 responds more robustly than *XTH3*. This indicates that the *XTH* gene members within paralogous pairs may have undergone functional differentiation, contributing to distinct biological functions. Additionally, some genes exhibit specific responses to individual stressors; for instance, *XTH12* is induced solely under drought stress conditions, suggesting its potential involvement in drought response (Fig. 5).

### Co-expression network of *XTHs* in poplar

**Figure 6:**
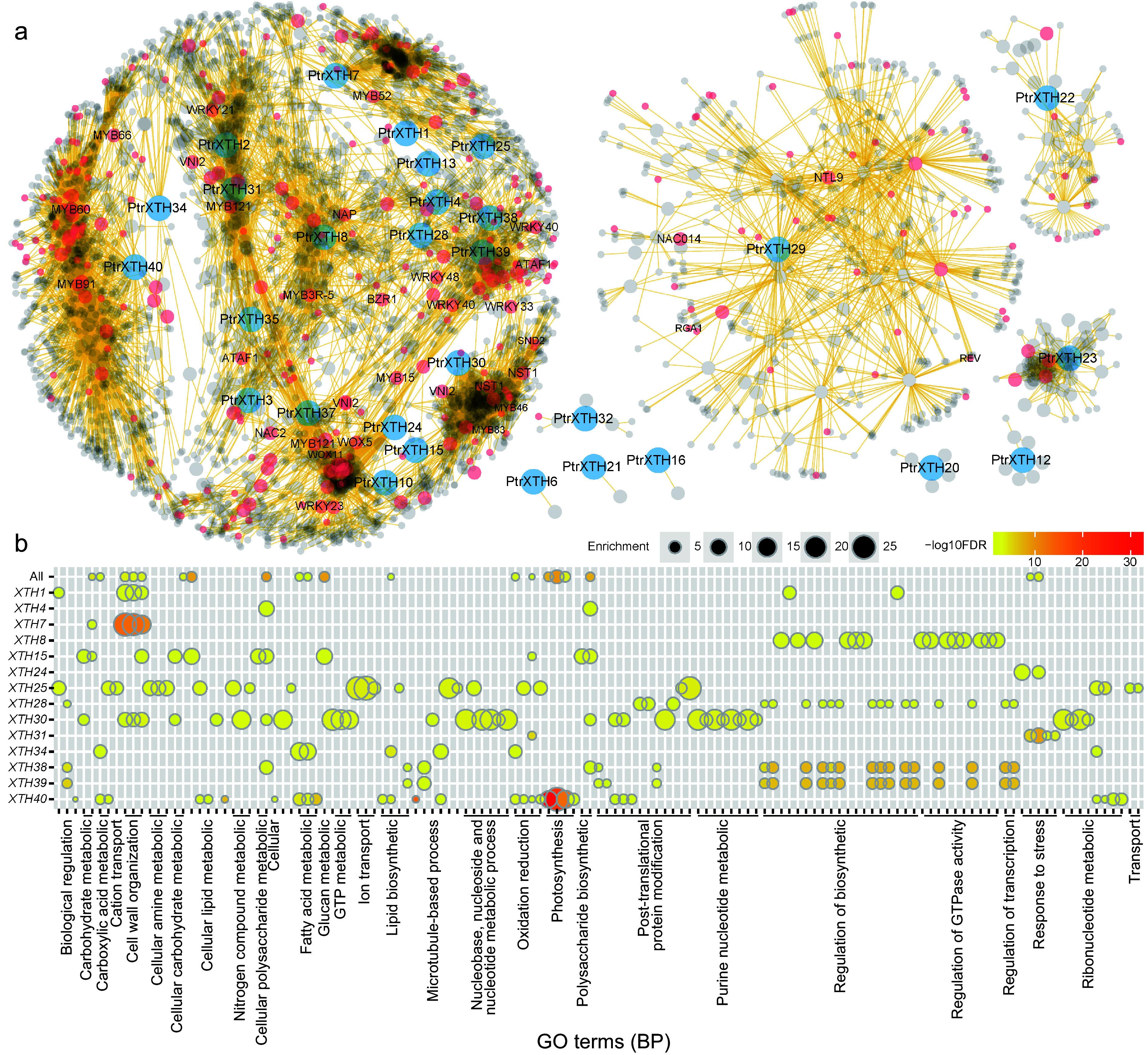
The co-expression network of *PtrXTHs*. (a) Co-expression network of *PtrXTHs*. Blue, red, and grey nodes represent *PtrXTHs*, transcription factors, and other genes, respectively. (b) Gene ontology (GO) enrichment analysis of the co-expression sub-networks of 14 *PtrXTHs* on biological process (BP). The color gradient from yellow to red signifies the −log10 transformed FDR-corrected *p* value, while node size reflects the rich factor in the respective GO terms.

The co-expression network provides insights into the potential functions and evolutionary divergences of genes. To explore the potential functions and evolutionary divergences of *XTH* family members in poplar, a co-expression network of *PtrXTHs* based on the comprehensive poplar genome expression atlas database was constructed (Fig. 6a). A total of 4,270 genes were co-expressed with 29 *PtrXTHs* genes, with varying numbers of co-expressed genes for different *PtrXTHs* ranging from 2 to 953. Six genes (*PtrXTH6*, *PtrXTH12*, *PtrXTH16*, *PtrXTH20*, *PtrXTH21*, and *PtrXTH32*) exhibited limited co-expression with 2, 8, 2, 6, 2, and 8 genes, respectively, forming six independent subnetworks (Fig. 6a & Supplementary Table S5). In contrast, *PtrXTH22*, *PtrXTH23*, and *PtrXTH29* each co-expressed with 84, 67, and 499 genes, forming intricate independent subnetworks. The remaining 20 *PtrXTHs* co-express with 3,741 genes, including *BZR*, *WRKY*, *WOX,* and others, composing a complex co-expression network (Fig. 6b). Genes with similar expression patterns in this network, particularly those functionally related, may share comparable functionalities.

To delve deeper into the potential biological processes involving *XTHs*, a Gene Ontology (GO) enrichment analysis was constructed based on co-expressed genes (Fig. 6b, Supplementary Figs S3−S5, & Supplementary Table S6). Among the 20 *PtrXTH* gene subnetworks, 14 *PtrXTHs* exhibited enrichment in various GO terms, primarily associated with biological processes. Within the same subnetwork, *PtrXTH38* and *PtrXTH39* demonstrated noticeable enrichment, particularly related to the biological regulation, lipid biosynthetic post-translation protein modification, regulation of biosynthetics, regulation of GTPase activity and regulation of transcription. A plausible explanation is that *PtrXTH38* and *PtrXTH39* paralogous pair form tandem duplication, indicating potential functional proximity. These findings suggest that *PtrXTH38* and *PtrXTH39* play analogous roles in a certain biological process, potentially pivotal in shared biological pathways. This provides clues for further comprehending the functions of these genes in plant growth and development (Fig. 6b).

### Analysis of PagXTH12 protein structure and activity

**Figure 7:**
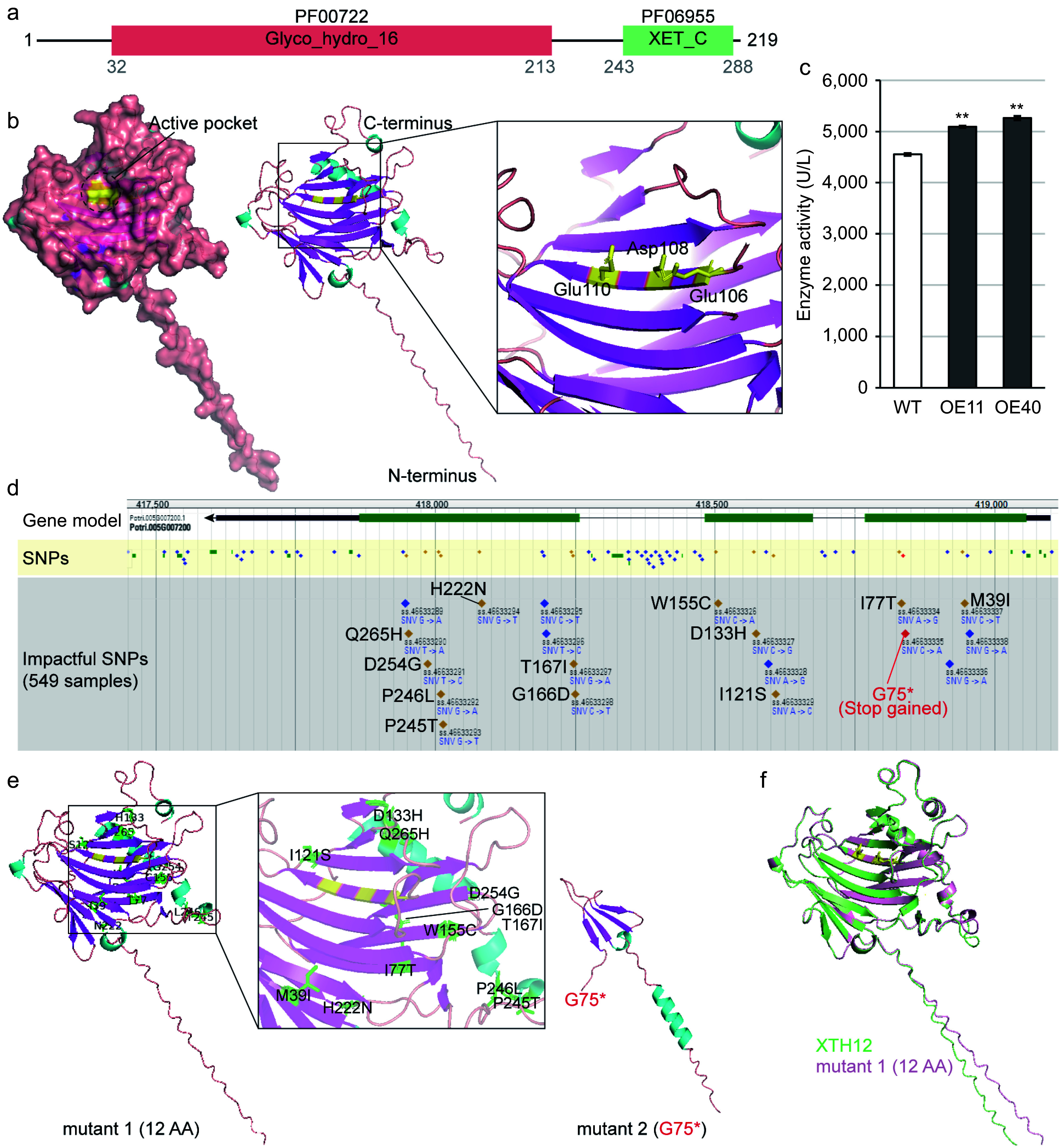
Analysis of PagXTH12 protein structure and activity. (a) The conserved protein domain of PagXTH12. (b) Structural model of PagXTH12, with three amino acids (Glu106, Asp108, and Glu110 marked in yellow) identified as catalytic active sites located at the center of the active pocket. (c) Analysis of *in vivo* XTH catalytic activity in two *PagXTH12*-overexpressing poplar lines (OE11 and OE40) and wild-type control (WT). ** *p* < 0.01. (d) Single nucleotide polymorphisms (SNPs) within the *XTH12* gene region among a population of 549 individuals of *Populus trichocarpa*. (e) Protein structures of two proposed mutants of PagXTH12, mutant 1 contains 12 non-synonymous mutation amino acids (marked in green), while mutant 2 has a premature termination codon at position 75. (f) Alignment results between PagXTH12 and its proposed mutant 1.

The functional domains of *Pag*XTH12 were analyzed and two conserved structural regions identified within its protein: the glycosyl hydrolases family 16 domain (PF00722) from 32 to 213 aa, and the Xyloglucan endo-transglycosylase (XET) C-terminus domain (PF06955) between 243 and 288 aa (Fig. 7a). Utilizing AlphaFold for protein structure prediction, it was found that PagXTH12 exhibits a groove-like structure, with a central region corresponding to the previously reported conserved active site domain ExDxE of the GH16 family XTH. This active site is represented by the residues Glu106, Asp108, and Glu110, forming a distinct catalytic pocket (Fig. 7b). To confirm the catalytic activity of PagXTH12, transgenic poplar lines that overexpress *PagXTH12* were created. Among the 51 transgenic lines, two high-expression lines OE11 and OE40 through qRT-PCR analysis were used for enzyme activity assays and subsequent experiments (Supplementary Fig. S6). Compared to wild-type controls, the XTH catalytic activity in these lines increased by 11.9% and 15.6%, respectively (Fig. 7c). To further elucidate the conservation of the active site in XTH12, single nucleotide polymorphisms (SNPs) within the *XTH12* gene among a population of 549 independent *Populus trichocarpa* individuals were investigated. Nineteen impactful SNPs were identified in the coding region of the *XTH12* gene, including 12 non-synonymous SNPs, six synonymous SNPs, and one premature termination codon gained SNP. Notably, among the non-synonymous mutations, the three active residues (Glu106, Asp108, and Glu110) showed no mutations, indicating that the integrity of this gene's catalytic site is likely essential for its functionality (Fig. 7d). Subsequently, structural simulations were conducted on two hypothesized mutated sequences: mutant 1, which contained the 12 non-synonymous mutations, and mutant 2, which included a premature termination at the 75^th^ amino acid position. Analysis revealed that the positions of amino acid changes due to the non-synonymous mutations were localized outside the active pocket of the protein, suggesting that these mutations would not impair the protein's catalytic activity (Fig. 7e). Furthermore, comparing the protein structure of XTH12 with that of mutant 1, despite the presence of 12 amino acid changes, indicated that the overall protein structure remained stable (Fig. 7f). These results collectively suggest that the XTH12 protein likely possesses a conserved biological function.

### Overexpressing the *PagXTH12* gene can improve drought resistance

**Figure 8:**
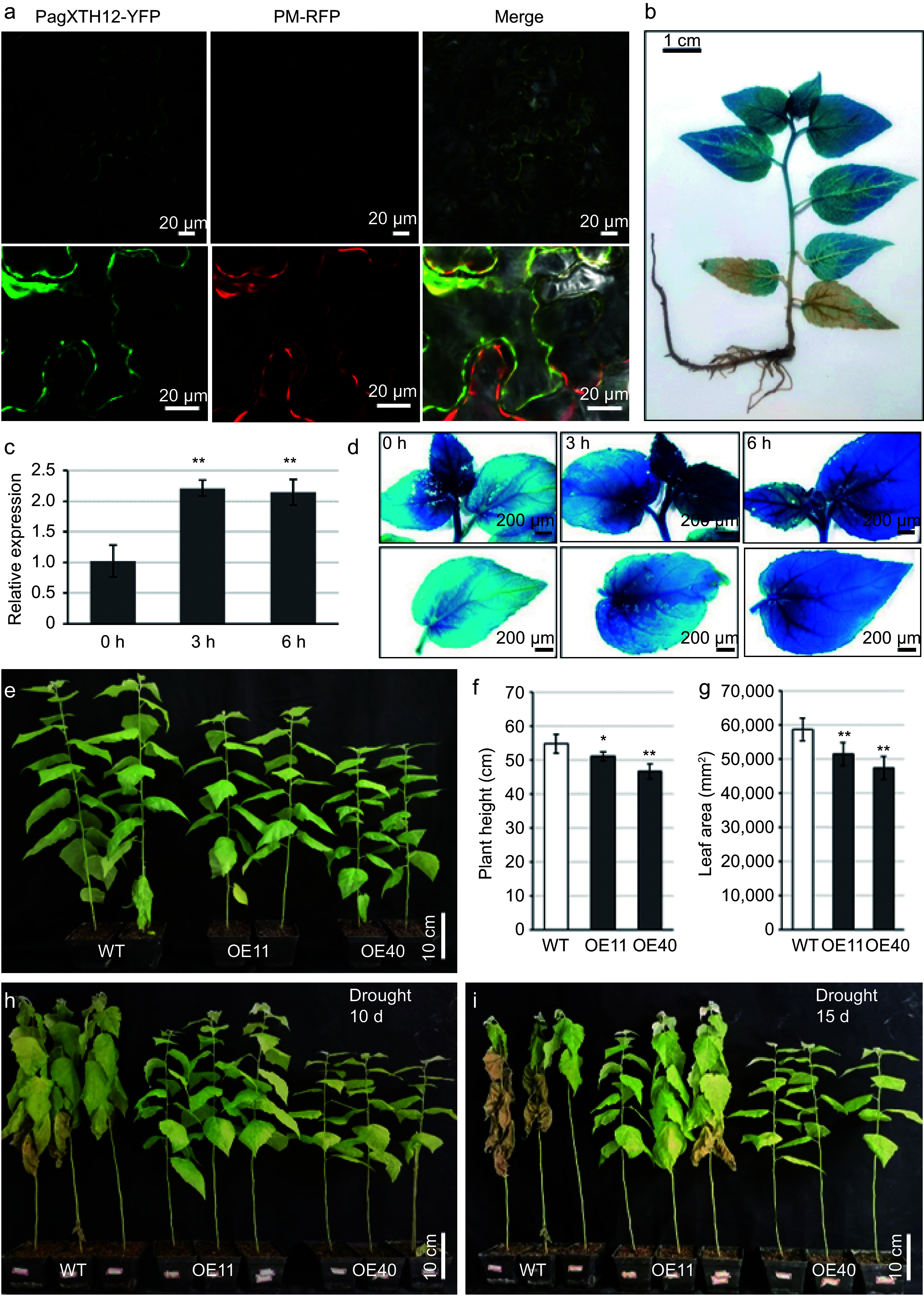
*PagXTH12* responds to drought stress and enhances drought resistance in transgenic poplar. (a) Subcellular localization of the PagXTH12-YFP fusion protein in the lower epidermal cells of leaves in *Nicotiana benthamiana*. (b) GUS staining of Pro*PagXTH12::GUS* transgenic poplar. (c) Expression of *PagXTH12* under 20% PEG6000 simulated drought stresses at 0 h, 3 h, and 6 h, the data are presented as mean ± SD. ** *p* < 0.01. (d) The GUS staining of Pro*PagXTH12::GUS* transgenic poplar under PEG-simulated drought stress conditions shows that the GUS signal intensity increases with prolonged drought treatment duration. (e)−(g) Under well-watered conditions, the overexpression of *PagXTH12* inhibits plant height and biomas. After 90 d of cultivation, the (f) plant height, and (h) total leaf area of the overexpression lines OE11 and OE40 were significantly lower than the control. * *p* < 0.05, ** *p* < 0.01. (h), (i) After 10 and 15 d of natural drought stress, the *PagXTH12* overexpression lines OE11 and OE40 exhibited significantly improved drought resistance compared to the control.

To further elucidate the function of *XTH12*, experimental validation of its localization was conducted at both the subcellular and tissue levels. Subcellular localization analysis revealed that the PagXTH12 protein is likely localized to the cell wall and cytoplasm, a finding confirmed through plasmolysis experiments (Fig. 8a). Additionally, the promoter of *PagXTH12* was cloned and transformed into poplar trees. GUS staining of Pro*PagXTH12::GUS* seedlings demonstrated expression of *PagXTH12* in the apical region, leaves, and stems of the plants (Fig. 8b).

To assess the response of *PagXTH12* to drought stress, qRT-PCR was performed to measure its gene expression under PEG6000-induced drought conditions. The results indicated that *PagXTH12* expression was significantly induced after 3 and 6 h of simulated drought treatment (Fig. 8c). Subsequently, Pro*PagXTH12::GUS* transgenic seedlings were subjected to drought stress, observing that GUS staining activity was significantly induced with increasing drought duration. These findings suggest that the expression of *PagXTH12* is directly induced by drought stress, indicating its potential involvement in the drought stress response (Fig. 8d).

Subsequently, *PagXTH12* overexpression transgenic poplar trees were developed to investigate the biological function of *PagXTH12*. Among the more than ten transgenic lines obtained, the two lines with the highest expression levels (OE11 and OE40) were selected for further functional studies. After three months of cultivation under normal watering conditions, the transgenic plants overexpressing *PagXTH12* exhibited phenotypes characterized by reduced height, decreased total leaf area, and lower biomass (Fig. 8e−g). Upon cessation of watering and subjecting the plants to natural drought stress, the wild-type (WT) plants began to wilt by the 10^th^ d, while the two overexpression lines maintained relatively good growth (Fig. 8h). By the 15^th^ d of drought stress, most leaves of the WT plants had withered, whereas the leaves of the *PagXTH12* overexpression lines still retained a certain level of vitality (Fig. 8i). These results indicate that *PagXTH12* enhances drought stress resistance in transgenic plants.

## Discussion

The poplar serves as a crucial model plant and energy resource, intimately linked to human production and daily life, playing a pivotal role in various applications. The *XTH* gene family is involved in the hydrolysis and transfer of hemicellulose xyloglucan chains within the cell wall, exerting an influence on the formation of the cell wall^[34]^.

In this study, a total of 71 *PagXTH* genes were identified in two subgenomes of the '84K' poplar, comprising 38 members in subgenome A and 33 in subgenome G. Notably, the number of members in each subgenome of '84K' poplar is less than that observed in *P. trichocarpa*, which has 41 *XTHs*. The estimated genome size of *P. trichocarpa* is 391.57 Mb^[35]^, while the sizes of subgenome A and subgenome G of '84K' poplar are approximately 356 Mb and 354 Mb, respectively^[36]^. The size of a gene family is positively correlated with genome size. In addition, genome-wide duplication events contribute to the accumulation of members within the gene family^[37]^. During evolution, different species of poplar genomes experience gene loss or rearrangement. Comparative genomic analyses indicate that subgenome G harbors more paralogous genes in relation to *P. trichocarpa* than subgenome A, resulting in a greater retention of genes in subgenome A. Subgenome G exhibits a higher incidence of single-copy gene loss, and studies suggest that gene dosage can influence gene expression levels, implying potential functional consequences for the lost genes^[38]^. The number of identified genes is contingent upon the completeness of the genome assembly. For example, the 3.0 version of the *P. trichocarpa* genome assembly identified 43 *XTHs* on scaffolds, but subsequent updates may have reduced this number to 41. Future improvements in genome completeness, facilitated by long-read sequencing and the utilization of Telomere-to-Telomere (T2T) genome assemblies^[39]^, are expected to improve the accuracy of gene identification.

Colinearity analysis was employed to examine the evolutionary patterns within the *XTH* gene family of *P. trichocarpa* and '84K' poplar. Additionally, the promoter sequence similarities among paralogous gene pairs were compared. Paralogous pairs with high promoter similarity, such as W1 (*XTH1/9*) and W9 (*XTH29/40*), exhibited relatively similar expression levels across different tissues in poplar. Particularly, W9 demonstrates consistent expression changes across various stress treatments. The results implied that promoter differences indeed influence gene expression. The promoter region, as a key component of the gene regulatory region, plays a pivotal role in orchestrating the initiation of gene transcription. The expression of homologous genes that are similar is associated with a greater presence of similar promoter regions compared to the expression of homologous genes that are dissimilar^[40]^.

Through the analysis of expression patterns under various stress conditions, *XTH12* was identified as a potential drought-responsive gene due to its significant expression increase during prolonged drought. The promoter region of *PagXTH12* contains numerous stress-related *cis*-acting elements, including the MBS and DRE *cis*-acting elements that are associated with drought stress. Furthermore, *PagXTH12* features an ABRE *cis*-acting elements, which is involved in the abscisic acid (ABA) signaling pathway, a core component of plant responses to drought and salt stress^[41]^. Drought stress triggers enhanced drought resistance through hormone-induced ABA accumulation and downstream signaling activation across various plant organs^[42]^. There may also be a connection between cell wall biosynthesis and ABA-dependent regulation^[43]^. Many studies across different plant species indicate that members of the *XTH* gene family play a significant role in responding to osmotic stresses. For instance, drought stress affects the expression of *XTH11* and *XTH29* in Arabidopsis roots^[44]^, and overexpression of *CaXTH3* has been shown to enhance drought tolerance in tomato^[45]^.

The XTH family possesses both hydrolase (XTH) and transglycosylase (XEH) functions, with most XTHs having detailed kinetic data exhibiting strict XET activity^[46]^. The predictive results of the protein structure indicate that XTH12 contains a conserved active catalytic site, ExDxE, which is consistent with motif predictions. In the poplar population, variations in the sequence do not affect the active site or the protein structure, suggesting the conservation and significance of this gene's function. Compared to the WT, the XTH catalytic activities of OE11 and OE40 were increased by 11.9% and 15.6%, respectively. After the increase in XTH activity, the biomass of OE11 and OE40 significantly decreased. The role of *XTHs* in regulating plant growth is complex, as evidenced by various studies. For instance, overexpression of *AtXTH31* or *AtXTH32* does not result in significant growth phenotypes^[47]^. Similarly, overexpression of *XTH22* (*TCH4*) does not cause noticeable changes in growth under normal conditions in *Arabidopsis*, but under low boron (low B) stress, overexpression leads to growth inhibition^[11]^. In contrast, overexpression of *BcXTH1* promotes growth^[48]^. These findings suggest that plant growth is regulated by a more intricate network, with changes in cell wall structure potentially affecting plant growth and development differently under various environmental conditions. Future studies could employ tissue-specific promoters to drive the expression of XTH genes, which would allow for a more detailed examination of how *XTHs* regulate specific cell types and their subsequent impact on plant growth and development. In general, there exists a trade-off between growth and resistance in plants; those that grow well may exhibit lower resistance, while those with strong resistance may have reduced growth^[49]^. Following the overexpression of *PagXTH12*, biomass decreases, characterized by reduced plant height and total leaf area, potentially due to changes in the activity of wall-modifying enzymes involved in cell expansion, including expansins (EXPA/B) and xyloglucan endotransglycosylases/hydrolases (XTH)^[50]^. Drought is directly related to the loss of water from leaves; under drought conditions, plants may reduce leaf area to decrease water evaporation, thereby enhancing drought resistance^[51]^. The subcellular localization prediction for PagXTH12 indicated its presence in both the cell wall and cytoplasm, indicating its functional presence in both the cell wall and cytoplasm. Under drought stress, plants typically initiate stomatal closure as a primary defense mechanism to prevent water loss. The *XTH* gene plays a crucial role in altering cell wall elongation and enhancing drought resistance^[52]^. Consequently, it can be hypothesized that, upon encountering drought stress, the increased enzymatic activity of PagXTH12(A) may help the poplar respond to drought by enhancing the regulation of cell wall modification.

In summary, an in-depth investigation of the *XTH* gene family in poplar not only contributes to unraveling the molecular mechanisms underlying plant cell wall regulation but also provides scientific foundations for the genetic improvement and sustainable utilization of poplar. *XTH12* stands out as a potential key gene in response to drought stress, suggesting that targeted modulation of its expression could pave the way for promising strategies in breeding drought-resistant trees through the regulation of cell wall modifications.

## Conclusions

In summary, this study identified a total of 41 *PtrXTH*s in the *P. trichocarpa* and 71 *PagXTH*s in '84K' poplar*,* respectively. Structural analysis, examination of promoter *cis*-acting elements, investigation of gene duplication events, assessment of promoter similarity, and analysis of expression patterns were conducted for this gene family. Additionally, *PagXTH12* was speculated to play a role in response to drought stress. The overexpression of *PagXTH12(A)* increased the enzymatic activity and enhanced drought resistance in the transgenic poplar. This study lays the groundwork for further research on the regulatory roles of the *XTH* gene family in the growth processes of poplar and their response to drought stress.

## SUPPLEMENTARY DATA

Supplementary data to this article can be found online.

## Data Availability

All data generated or analyzed during this study are included in this published article and its supplementary information files.
