# Supplementary material for: Identification of the xyloglucan endotransglycosylase/hydrolase genes and the role of PagXTH12 in drought resistance in poplar
Source: For Res (Fayettev). 2024 Dec 31;4:e039. doi: 10.48130/forres-0024-0036 (PMC11870306; doi:10.48130/forres-0024-0036)
Supplement: Supplementary file 1 — Supplementary data to this article can be found online. [file forres-0024-0036-S1.zip › 10.48130_forres-0024-0036-Suppl-TableS2.pdf]

Table S2. *XTH* gene ID in *Populus trichocarpa* (*Ptr*) and *Populus alba* × *glandulosa* (*Pag*)

P.trichocarpa		'84K' subgenome A		'84K' Subgenome G	
Gene ID	Gene name	Gene ID	Gene name	Gene ID	Gene name
Potri.001G071000	*PtrXTH1*	PopA01G074711	*PagXTH1(A)*	PopG01G002830	*PagXTH1(G)*
Potri.001G136100	*PtrXTH2*	PopA01G004173	*PagXTH2(A)*	PopG01G089278	*PagXTH2(G)*
Potri.002G060400	*PtrXTH3*	PopA02G005521	*PagXTH3(A)*	PopG02G065437	*PagXTH3(G)*
Potri.002G060500	*PtrXTH4*	PopA02G005519	*PagXTH4(A)*	PopG02G065438	*PagXTH4(G)*
Potri.002G153200	*PtrXTH5*	PopA02G012191	*PagXTH5(A)*	PopG02G030954	*PagXTH5(G)*
Potri.002G236200	*PtrXTH6*	PopA02G023780	*PagXTH6(A)*	PopG02G015923	*PagXTH6(G)*
Potri.002G244200	*PtrXTH7*	PopA02G023990	*PagXTH7(A)*	PopG02G015825	*PagXTH7(G)*
Potri.003G097300	*PtrXTH8*	PopA03G050272	*PagXTH8(A)*	PopG03G010661	*PagXTH8(G)*
Potri.003G159700	*PtrXTH9*	PopA03G020147	*PagXTH9(A)*	PopG03G013929	*PagXTH9(G)*
Potri.004G021000	*PtrXTH10*	PopA04G018283	*PagXTH10(A)*	PopG04G023264	*PagXTH10(G)*
Potri.005G007200	*PtrXTH12*	PopA05G016860	*PagXTH12(A)*	n.a.	n.a.
Potri.005G201200	*PtrXTH13*	PopA05G056354	*PagXTH13(A)*	PopG05G008459	*PagXTH13(G)*
Potri.006G071200	*PtrXTH14*	PopA06G061600	*PagXTH14(A)*	PopG06G075982	*PagXTH14(G)*
Potri.006G122900	*PtrXTH15*	PopA06G085762	*PagXTH15(A)*	PopG06G051718	*PagXTH15(G)*
Potri.006G160700	*PtrXTH16*	PopA06G079417	*PagXTH16(A)*	PopG06G051233	*PagXTH16(G)*
Potri.006G169900	*PtrXTH17*	PopA06G064752	*PagXTH17(A)*	PopG06G053835	*PagXTH17(G)*
Potri.006G170001	*PtrXTH18*	PopA06G064753	*PagXTH18(A)*	PopG06G053834	*PagXTH18(G)*
Potri.006G170100	*PtrXTH19*	n.a.	n.a.	n.a.	n.a.
Potri.007G008500	*PtrXTH20*	PopA07G022723	*PagXTH20(A)*	PopG07G062649	*PagXTH20(G)*
Potri.008G138400	*PtrXTH21*	PopA08G086532	*PagXTH21(A)*	PopG08G046407	*PagXTH21(G)*
Potri.009G006600	*PtrXTH22*	PopA09G083696	*PagXTH22(A)*	PopG09G027710	*PagXTH22(G)*
Potri.009G083800	*PtrXTH23*	PopA09G015395	*PagXTH23(A)*	PopG09G077522	*PagXTH23(G)*
Potri.010G102300	*PtrXTH24*	PopA10G048486	*PagXTH24(A)*	PopG10G000227	*PagXTH24(G)*
Potri.011G025800	*PtrXTH25*	PopA11G091347	*PagXTH25(A)*	n.a.	n.a.
Potri.011G077320	*PtrXTH26*	PopA11G056756	*PagXTH26(A)*	n.a.	n.a.
Potri.011G077380	*PtrXTH27*	n.a.	n.a.	n.a.	n.a.
Potri.013G005700	*PtrXTH28*	PopA13G054411	*PagXTH28(A)*	PopG13G022225	*PagXTH28(G)*
Potri.013G152400	*PtrXTH29*	PopA13G031118	*PagXTH29(A)*	PopG13G072741	*PagXTH29(G)*
Potri.014G115000	*PtrXTH30*	PopA14G044010	*PagXTH30(A)*	PopG14G051007	*PagXTH30(G)*
Potri.014G140300	*PtrXTH31*	PopA14G045470	*PagXTH31(A)*	PopG14G000593	*PagXTH31(G)*
Potri.014G146100	*PtrXTH32*	PopA14G045767	*PagXTH32(A)*	PopG14G000662	*PagXTH32(G)*
Potri.014G152700	*PtrXTH33*	n.a.	n.a	n.a.	n.a.
Potri.016G098600	*PtrXTH34*	PopA16G028679	*PagXTH34(A)*	PopG16G068625	*PagXTH34(G)*
Potri.018G084300	*PtrXTH35*	PopA18G018913	*PagXTH35(A)*	PopG18G080149	*PagXTH35(G)*
Potri.018G094800	*PtrXTH36*	PopA18G018794	*PagXTH36(A)*	PopG18G080130	*PagXTH36(G)*
Potri.018G094900	*PtrXTH37*	PopA18G018793	*PagXTH37(A)*	PopG18G080131	*PagXTH37(G)*
Potri.018G095100	*PtrXTH38*	PopA18G018789	*PagXTH38(A)*	PopG18G080133	*PagXTH38(G)*
Potri.018G095200	*PtrXTH39*	PopA18G018791	*PagXTH39(A)*	n.a.	n.a.
Potri.019G125000	*PtrXTH40*	PopA19G055144	*PagXTH40(A)*	PopG19G084027	*PagXTH40(G)*
Potri.005G201250	*PtrXTH43*	PopA05G056355	*PagXTH43(A)*	n.a.	n.a.
Potri.009G163850	*PtrXTH44*	PopA09G077102	*PagXTH44(A)*	PopG09G011699	*PagXTH44(G)*
