# Supplementary material for: Identification of the xyloglucan endotransglycosylase/hydrolase genes and the role of PagXTH12 in drought resistance in poplar
Source: For Res (Fayettev). 2024 Dec 31;4:e039. doi: 10.48130/forres-0024-0036 (PMC11870306; doi:10.48130/forres-0024-0036)
Supplement: Supplementary file 1 — Supplementary data to this article can be found online. [file forres-0024-0036-S1.zip › 10.48130_forres-0024-0036-Suppl-TableS3.pdf]

Table S3. Characteristics of *XTHs* of hybrid poplar '84K'.

Gene Name	Gene ID	Chromosome location	AA	MW(Da)	PI	CDS(bp)	Subcellular localization
PagXTH1(A)	Pop_A01G004173	chrA01(-):37831133-37833946	351	40852.28	9.08	1056	Cell wall
PagXTH2(A)	Pop_A01G074711	chrA01(-):43380918-43382981	293	33989.46	8.45	882	Cell wall. Cytoplasm.
PagXTH3(A)	Pop_A02G023990	chrA02(-):1628047-1629881	272	31012.32	7.14	819	Cell wall
PagXTH4(A)	Pop_A02G023780	chrA02(-):2297892-2300258	288	32980.3	9.24	867	Cell wall. Cytoplasm.
PagXTH5(A)	Pop_A02G012191	chrA02(-):11242935-11244005	170	19160.6	5.13	513	Cell wall
PagXTH6(A)	Pop_A02G005519	chrA02(+):17974145-17975351	294	33490.04	9.27	885	Cell wall. Cytoplasm.
PagXTH7(A)	Pop_A02G005521	chrA02(+):17977241-17978291	289	32601.89	7.56	870	Cell wall. Cytoplasm.
PagXTH8(A)	Pop_A03G020147	chrA03(+):4891416-4893760	298	34791.56	8.4	897	Cell wall. Cytoplasm.
PagXTH9(A)	Pop_A03G050272	chrA03(+):9231032-9234445	348	40397.71	8.71	1047	Cell wall
PagXTH10(A)	Pop_A04G018283	chrA04(-):18915927-18917647	279	32298.07	4.74	840	Cell wall
PagXTH12(A)	Pop_A05G056355	chrA05(-):4094702-4095869	330	37191.45	9.02	993	Cell wall. Cytoplasm.
PagXTH13(A)	Pop_A05G056354	chrA05(-):4104542-4105601	294	33321.63	8.47	885	Cell wall. Cytoplasm.
PagXTH14(A)	Pop_A05G016860	chrA05(+):20719418-20720614	291	33029	4.54	876	Cell wall. Cytoplasm.
PagXTH15(A)	Pop_A06G064752	chrA06(+):9069411-9070519	287	32666.68	6.65	864	Cell wall. Cytoplasm.
PagXTH16(A)	Pop_A06G064753	chrA06(+):9080027-9081133	287	32709.71	7.04	864	Cell wall. Cytoplasm.
PagXTH17(A)	Pop_A06G079417	chrA06(+):10588797-10590497	291	33163.31	9.14	876	Cell wall
PagXTH18(A)	Pop_A06G085762	chrA06(+):14455149-14457552	330	37856.13	9.36	993	Cell wall
PagXTH20(A)	Pop_A06G061600	chrA06(-):18650084-18651141	269	29898.41	8.25	810	Cell wall. Cytoplasm.
PagXTH21(A)	Pop_A07G022723	chrA07(+):13192966-13194223	296	34089.82	7.08	891	Cell wall
PagXTH22(A)	Pop_A08G086532	chrA08(-):8064453-8067051	236	27216.36	6.45	711	Cell wall
PagXTH23(A)	Pop_A09G015395	chrA09(+):5210154-5211315	294	34398.86	9.14	885	Cell wall
PagXTH24(A)	Pop_A09G083696	chrA09(+):11169772-11171821	294	33181.17	6.3	885	Cell wall
PagXTH25(A)	Pop_A10G048486	chrA10(+):9994489-9996718	336	38242.91	6.21	1011	Cell wall
PagXTH26(A)	Pop_A11G056756	chrA11(-):9502121-9516438	518	59586.03	9.29	1557	Cell wall
PagXTH28(A)	Pop_A11G091347	chrA11(-):14728747-14730434	272	31863.62	4.7	819	Cell wall
PagXTH29(A)	Pop_A13G031118	chrA13(+):688926-691819	329	37349.17	5.94	990	Cell wall
PagXTH30(A)	Pop_A13G054411	chrA13(-):13936126-13937594	370	41373.56	6.59	1113	Cell wall. Cytoplasm.
PagXTH31(A)	Pop_A14G045767	chrA14(+):8642735-8644436	272	30997.07	9.11	819	Cell wall. Cytoplasm.
PagXTH32(A)	Pop_A14G045470	chrA14(-):9097957-9099330	240	27371.06	6.58	723	Cell wall. Cytoplasm.
PagXTH34(A)	Pop_A14G044010	chrA14(-):10881361-10882658	312	34941.33	6.66	939	Cell wall
PagXTH35(A)	Pop_A16G028679	chrA16(+):6308151-6310455	294	33943.51	9.46	885	Cell wall
PagXTH36(A)	Pop_A18G018789	chrA18(-):3799291-3801931	349	39514.25	5.03	1050	Cell wall. Cytoplasm.
PagXTH37(A)	Pop_A18G018791	chrA18(-):3805220-3806258	286	31977.75	4.91	961	Cell wall. Cytoplasm.
PagXTH38(A)	Pop_A18G018793	chrA18(+):3815307-3816391	291	33037.11	5.59	876	Cell wall. Cytoplasm.
PagXTH39(A)	Pop_A18G018794	chrA18(+):3820971-3822550	323	36650.93	8.36	972	Cell wall. Cytoplasm.
PagXTH40(A)	Pop_A18G018913	chrA18(-):4972888-4974565	291	33195.35	8.93	876	Cell wall
PagXTH43(A)	Pop_A19G055144	chrA19(-):521586-524048	296	33729.01	5.78	891	Cell wall
PagXTH44(A)	Pop_A09G077102	chrA09(-):407926-408813	295	33177.77	5.43		Cell wall
PagXTH1(G)	Pop_G01G089278	chrG01(-):38048006-38051012	351	40872.27	9.08	1056	Cell wall
PagXTH2(G)	Pop_G01G002830	chrG01(-):43855307-43858312	293	33953.45	8.45	882	Cell wall. Cytoplasm.
PagXTH3(G)	Pop_G02G015825	chrG02(-):1699033-1700885	272	31012.32	7.72	819	Cell wall
PagXTH4(G)	Pop_G02G015923	chrG02(-):2444280-2446404	289	33049.41	9.24	870	Cell wall. Cytoplasm.

**Gene Name**	**Gene ID**	**Chromosome location**	**AA**	**MW(Da)**	**PI**	**CDS(bp)**	**Subcellular localization**
PagXTH5(G)	Pop_G02G030954	chrG02(-):11277616-11278736	213	24066.9	4.77	642	Cell wall
PagXTH6(G)	Pop_G02G065438	chrG02(+):18472956-18474328	294	33437.97	9.36	885	Cell wall. Cytoplasm.
PagXTH7(G)	Pop_G02G065437	chrG02(+):18476176-18477609	289	32602.94	8.1	870	Cell wall. Cytoplasm.
PagXTH8(G)	Pop_G03G013929	chrG03(+):5635456-5637787	289	32602.94	8.1	885	Cell wall. Cytoplasm.
PagXTH9(G)	Pop_G03G010661	chrG03(+):10517014-10520862	349	40452.76	8.59	1050	Cell wall
PagXTH10(G)	Pop_G04G023264	chrG04(-):19405895-19407830	293	34011	4.85	882	Cell wall
PagXTH13(G)	Pop_G05G008459	chrG05(-):4022435-4030437	294	33235.54	8.83	885	Cell wall. Cytoplasm.
PagXTH14(G)	Pop_G06G053834	chrG06(+):8921018-8928046	409	45308.2	8	1230	Cell wall. Cytoplasm.
PagXTH15(G)	Pop_G06G053835	chrG06(+):8931321-8932386	287	32730.64	6.38	864	Cell wall. Cytoplasm.
PagXTH16(G)	Pop_G06G051233	chrG06(+):10403665-10405365	291	33177.33	9.14	876	Cell wall
PagXTH17(G)	Pop_G06G051718	chrG06(+):14552375-14554550	294	34006.64	9.64	885	Cell wall
PagXTH18(G)	Pop_G06G075982	chrG06(-):18931312-18932958	285	31816.72	8.2	858	Cell wall. Cytoplasm.
PagXTH20(G)	Pop_G07G062649	chrG07(+):13457322-13458964	296	34073.82	7.08	891	Cell wall
PagXTH21(G)	Pop_G08G046407	chrG08(-):11032561-11034891	337	38261.95	6.06	1014	Cell wall
PagXTH22(G)	Pop_G09G077522	chrG09(+):5279962-5281349	294	34354.81	9.14	885	Cell wall
PagXTH23(G)	Pop_G09G027710	chrG09(+):10980152-10981765	294	33155.13	6.3	885	Cell wall
PagXTH24(G)	Pop_G10G000227	chrG10(+):10073222-10076109	336	38187.83	6.07	1011	Cell wall
PagXTH28(G)	Pop_G13G072741	chrG13(+):643134-645678	208	24035.69	5.96	627	Cell wall
PagXTH29(G)	Pop_G13G022225	chrG13(-):14077989-14079060	293	32671.56	5.3	882	Cell wall. Cytoplasm.
PagXTH30(G)	Pop_G14G000662	chrG14(+):7308059-7309808	284	32284.54	8.95	855	Cell wall. Cytoplasm.
PagXTH31(G)	Pop_G14G000593	chrG14(-):7780697-7789498	470	54060.12	8.77	1413	Cell wall. Cytoplasm.
PagXTH32(G)	Pop_G14G051007	chrG14(-):9571741-9573319	312	34896.27	6.89	939	Cell wall
PagXTH34(G)	Pop_G16G068625	chrG16(+):4851250-4853931	294	33891.42	9.32	885	Cell wall
PagXTH35(G)	Pop_G18G080133	chrG18(-):4252180-4253219	286	31994.8	4.83	861	Cell wall. Cytoplasm.
PagXTH36(G)	Pop_G18G080131	chrG18(+):4264247-4265645	291	33009	5.55	876	Cell wall. Cytoplasm.
PagXTH37(G)	Pop_G18G080130	chrG18(+):4270677-4271727	287	32596.13	9.05	864	Cell wall. Cytoplasm.
PagXTH38(G)	Pop_G18G080149	chrG18(-):5749154-5750831	291	33152.28	8.93	876	Cell wall
PagXTH40(G)	Pop_G19G084027	chrG19(-):555704-559486	231	26355.37	5.57	696	Cell wall
PagXTH44(G)	Pop_G09G011699	chrG09(-):391349-392236	295	33232.86	5.64	888	Cell wall
