# Supplementary material for: Identification of the xyloglucan endotransglycosylase/hydrolase genes and the role of PagXTH12 in drought resistance in poplar
Source: For Res (Fayettev). 2024 Dec 31;4:e039. doi: 10.48130/forres-0024-0036 (PMC11870306; doi:10.48130/forres-0024-0036)
Supplement: Supplementary file 1 — Supplementary data to this article can be found online. [file forres-0024-0036-S1.zip › 10.48130_forres-0024-0036-Suppl-TableS1.pdf]

Table S1. qRT-PCR primers used for *PagXTH12* genes

Name	Gene ID	Label	Sequences (5'-3')
ACTIN		F	AAACTGTAATGGTCCTCCCTCCG
		R	GCATCATCACAATCACTCTCCGA
PagXTH12	PopA05G016860	F	CAAAGGCAACAGGGAGCAAC
		R	GGAGGCAGTGAAAGGAGCTT
